# Geographic atrophy: pathophysiology and current therapeutic strategies

**DOI:** 10.3389/fopht.2023.1327883

**Published:** 2023-12-05

**Authors:** Kalpana Rajanala, Farokh Dotiwala, Arun Upadhyay

**Affiliations:** Research and Development, Ocugen Inc., Malvern, PA, United States

**Keywords:** geographic atrophy, macular degeneration, drusen, inflammation, retinal biomarkers, gene therapy

## Abstract

Geographic atrophy (GA) is an advanced stage of age-related macular degeneration (AMD) that leads to gradual and permanent vision loss. GA is characterized by the loss of photoreceptor cells and retinal pigment epithelium (RPE), leading to distinct atrophic patches in the macula, which tends to increase with time. Patients with geographic atrophy often experience a gradual and painless loss of central vision, resulting in difficulty reading, recognizing faces, or performing activities that require detailed vision. The primary risk factor for the development of geographic atrophy is advanced age; however, other risk factors, such as family history, smoking, and certain genetic variations, are also associated with AMD. Diagnosis is usually based on a comprehensive eye examination, including imaging tests such as fundus photography, optical coherence tomography (OCT), and fluorescein angiography. Numerous clinical trials are underway, targeting identified molecular pathways associated with GA that are promising. Recent approvals of Syfovre and Izervay by the FDA for the treatment of GA provide hope to affected patients. Administration of these drugs resulted in slowing the rate of progression of the disease. Though these products provide treatment benefits to the patients, they do not offer a cure for geographic atrophy and are limited in efficacy. Considering these safety concerns and limited treatment benefits, there is still a significant need for therapeutics with improved efficacy, safety profiles, and better patient compliance. This comprehensive review discusses pathophysiology, currently approved products, their limitations, and potential future treatment strategies for GA.

## Introduction

**Figure 1:**
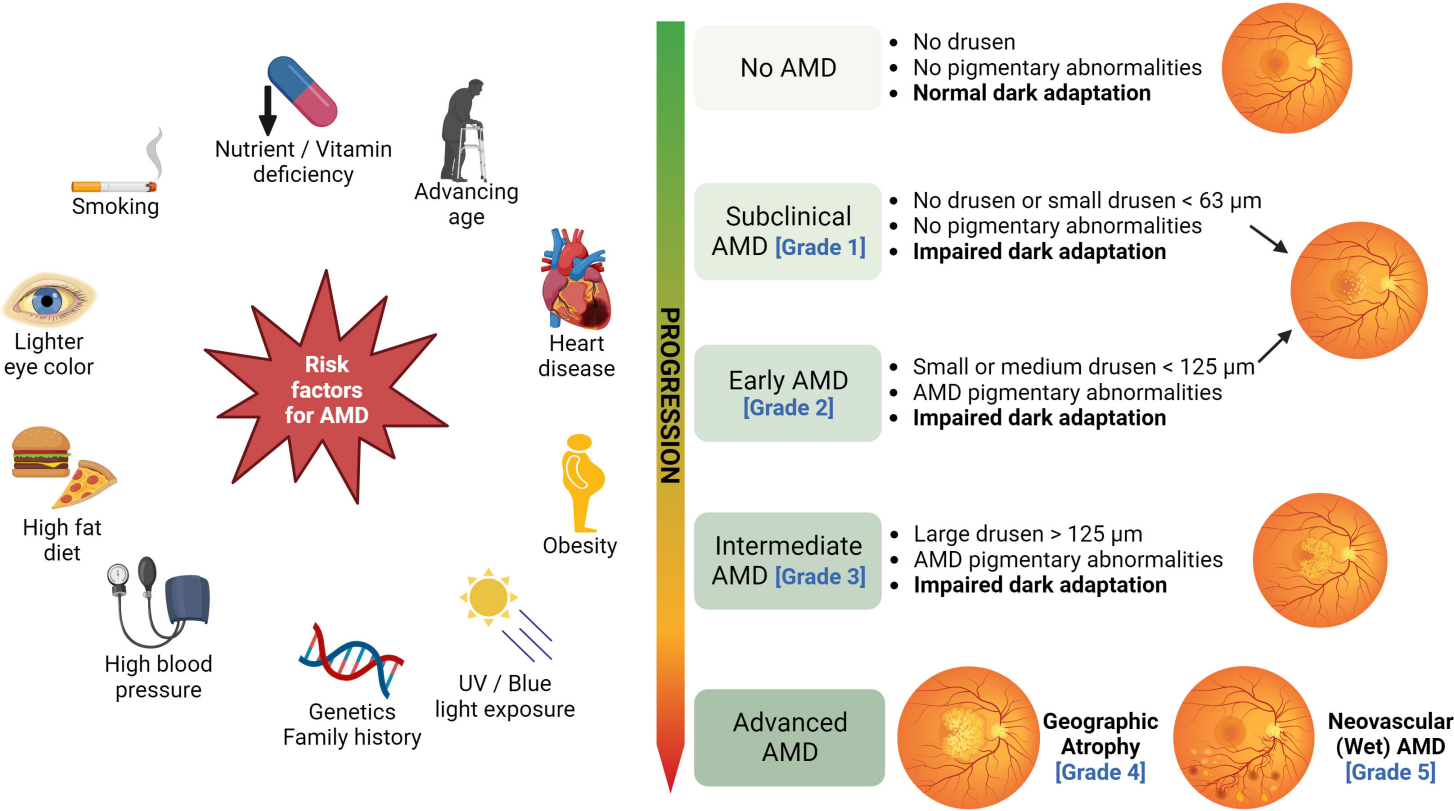
Risk factors and clinical stages of AMD: Several risk factors, such as cigarette smoking, advancing age, nutrient deficiency, high-fat diet, excessive UV/blue light exposure, obesity, and genetics contribute to the development of AMD. The clinical AMD staging system based on the size of drusen particles is illustrated on the right (figure generated using bioRender.com).

Age-related macular degeneration (AMD) is a progressive retinal neurodegenerative disease that involves the loss of photoreceptor cells and supportive retinal pigmented epithelial cells (RPE). The RPE has multiple functions, it acts as a blood-retina barrier, nourishes the photoreceptors, and is responsible for the phagocytosis of debris and wound healing (1). Several external and intrinsic risk factors (summarized in **Figure 1**) contribute to the pathophysiology of AMD, resulting in localized inflammation and neurodegeneration of the macula (the central part of the retina). The onset of the disease is characterized by the formation of lipid-rich extracellular deposits called drusen particles (2). AMD affects 28 million people worldwide and causes loss of vision in the population aged over 50 (3). The Clinical AMD staging system (4), based on the size of drusen particles, as identified by the color fundus photography, classifies the disease into the following grades: Grade 1, which has no drusen or few drusen deposits with sizes less than 63 µm. The presence of drusen between 63 to 124 µm is staged as Grade 2 or early AMD. Grade 3 or intermediate AMD is identified by the size of drusen ≥ 124 µm. The presence of drusen >125 µm is classified as Grade 4 or Geographic Atrophy (GA). Grade 5 is AMD with choroidal neovascularization (CNV), which is also known as wet or exudative AMD (**Figure 1**) (1, 4).

GA or dry/non-exudative AMD with or without foveal involvement is an advanced form of AMD, which affects over 8 million people globally, and it is known to cause permanent visual impairment (3, 5). The term “geographic” is used because the atrophic areas typically appear as well-defined, irregularly shaped lesions that may resemble the outlines of a map. These lesions can vary in size and may progress over time. The incidence of GA is more prevalent in the European population compared to the Asian, African, and Hispanic people (6, 7). Especially, GA incidence was shown to increase four-fold every ten years from the ages 50 to 80 in the European population (8, 9). GA affects the outermost layers of the retina, leading to a progressive decline in retinal pigment epithelial cells (RPE), photoreceptor cells, and reduced vessel density in the choriocapillaris (3, 10). The foveal region is located at the center of the macula, it comprises a high density of cone photoreceptors and is responsible for central and color vision. The rate of progression of GA varies according to the size and the location of the atrophy and the extent of foveal involvement (11, 12). GA lesions initially appear in the perifoveal macula, excluding the foveal center, and expand over time, usually within 1.5 to 2.4 years, to include the fovea (9, 12). Perifoveal atrophy causes impairment in reading, driving, and low-light vision, and atrophy extending to the foveal region can cause severe impairment in central vision (13).

Globally, the aging population is estimated to increase over the coming decades, and the incidence of GA is projected to rise (6). To date, intravitreal pegcetacoplan (Syfovre; Apellis Pharmaceuticals, Inc., approved in 2023) and Iveric Bio’s Izervay are commercially available in the United States to slow down the progression of GA. Therefore, there is an emerging need to discover new approaches to treat and manage this disease. This review discusses the risk factors, pathophysiology, and potential treatment strategies for GA.

## Diagnosis of geographic atrophy

### Visual acuity

GA is diagnosed by visual function tests to assess the extent of visual impairment. The best corrected visual acuity (BCVA) test utilizes the Snellen chart or the early treatment diabetic retinopathy study chart to measure how many letters a patient can read from a distance. Depending on the extent of foveal involvement, the patients may be able to read the letters and maintain visual acuity (VA). Reading speed can also be used to assess the progression of GA, where the number of correctly read words are quantified over a specific time. In a study, it was found that in patients with GA, the reading rate decreased significantly from 110 words per minute (wpm) at baseline to 51 wpm at two years (13). Low-luminance visual acuity (LLVA) measures visual function in low light by using a neutral density filter, which reduces the luminance by 2 log units and is conducted like BCVA (14). The difference between the patient’s BCVA and LLVA scores is termed low-luminance deficit (LLD), which is predictive of lesion enlargement in GA and subsequent vision loss (14, 15).

A patient’s central visual field can be examined by the Amsler grid, in which the graph lines can be distinguished accurately by the healthy eye. However, in patients with AMD, the lines may appear wavy, distorted, or blurred, and the grid may appear to have “holes,” dark areas, or scotomas, which are indicative of visual impairment (9).

Another visual function that is impaired in GA is dark adaptation. In the test to determine this function, the eye is exposed to a bright light to bleach most of the rhodopsin from rod and cone photoreceptors, and subsequently, the eye is stimulated with a bright light of ~420 nm wavelength against a pitch-black background. Delays in dark adaptation are observed in AMD patients with increasing severity of the disease determined by the presence of subretinal drusen deposits, drusen grade, and RPE pigment changes (16).

### Microperimetry

Microperimetry utilizes varying light intensities to stimulate different regions of the macula and is used to measure retinal sensitivity. It combines perimetry and retinal imaging to measure the sensitivity of the macula. It involves the projection of light stimuli on different points of the retina to generate a detailed “map” of the macular sensitivity to light and performs a fixation analysis at each moment of the examination. Microperimetry uses different technologies to assess residual visual function and functional vision. It has been used for the study of retinal macular disorders such as AMD, diabetic macular edema (DME), and macular dystrophies. It is specifically indicated for the early monitoring and diagnosis of pathologies affecting the macula and evaluating detailed vision and reading ability, along with other precision activities. A decrease in retinal sensitivity correlates with lesion enlargement and is associated with GA progression over time (17). Recently, automated perimetry was combined with scanning ophthalmoscopy to perform the structure-function analysis of GA (18). The correlation between the functional data and the anatomic morphology that can be generated by this technique aids in assessing the changes in GA lesion progression. In patients with GA, assessment of mean retinal sensitivity, pointwise or regional sensitivity, perilesional sensitivity, and fixation stability can characterize the severity of the disease (19).

The clinical features of macular degeneration or geographic atrophy can be characterized by the imaging tests described below.

### Color fundus photography

Retinal fundus photography provides two-dimensional (2D) color images of the retina as taken by a fundus camera. The images can be used to visualize abnormalities such as the presence of drusen, Lipofuscin granules, lipids, blood, scar tissue, and regions of atrophy (20). In Geographic atrophy, the drusen appear as round yellow lesions in the images, and the atrophic RPE can be distinguished by hypopigmentation (21). This method can be used to categorize drusen according to their size. However, the images obtained by FP lack depth, and they do not provide detailed quantitative information (21). Due to these limitations, this technique is often combined with other diagnostic methods for accuracy.

### Fundus autofluorescence

Another non-invasive imaging technique that is extensively popular in both clinical and research settings due to its ability to aid in the diagnosis and management of a variety of retinal disorders is fundus autofluorescence (FAF). It imparts better detection of exudative retinal diseases such as choroidal neovascularization, and a density map of lipofuscin, the predominant ocular fluorophore, in the retinal pigment epithelium. This provides information on the metabolic state and overall health of the RPE and, indirectly, the photoreceptor layer. Abnormal autofluorescence (AF) patterns on FAF imaging can act as markers for retinal disease. Since many retinal pathologies lead to RPE dysfunction and an accumulation of lipofuscin, abnormal AF patterns can help in the diagnosis and monitoring of retinal disease progression. FAF imaging is ideal for detecting orange pigment, which is sometimes subtle or near-invisible funduscopically, especially in deep or amelanotic lesions. FAF patterns may be linked to disease progression in patients with AMD and may thereby help clinicians determine appropriate therapeutic courses in the future. Fundus autofluorescence occurs due to drusen (lipid) deposits in the retinal pigment epithelium (22) comprising of lipids and fluorophores such as Lipofuscin (LF) (23), and A2E (N-retinylidene-N-retinylethanolamine) (24). Areas of atrophy in dry AMD appear as regions with reduced fluorescent signal due to the loss of RPE cells and photoreceptors and thus can be detected by FAF (25). The patterns of FAF associated with GA lesions can be correlated with the enlargement rate, with more diffuse hyper-autofluorescence changes generally representing a faster rate of progression (25). FAF can also be used to determine the size of GA lesions and the area of GA lesion progression. A recent meta-analysis implicated that determining the mean GA growth rate by FAF can be an important primary outcome measure for GA treatment trials (26). A novel quantitative FAF method (qAF) combines autofluorescence intensities measured by scanning ophthalmoscope with spatial information, which can be helpful in establishing structural correlations and allows for distinguishing changes related to disease progression (27). The limitations of FAF imaging include low autofluorescence signal compared to CFP, high image noise, low contrast, and interference from the anterior segment (28).

In summary, FAF is a valuable tool for clinicians in diagnosing and managing retinal diseases. It provides a density map of lipofuscin, detects abnormal patterns of autofluorescence, and helps visualize orange pigment. FAF imaging aids in the evaluation of prognosis in patients with age-related macular degeneration. However, there are some limitations to this imaging technique, including approximately two orders of magnitude lesser signal strength than the peak signal of fluorescein angiography. FAF imaging uses short-wavelength excitation light in the blue range, which limits the clear visualization of the central retina due to the absorption of blue light by macular pigment. This leads to limited visualization of the fovea and parafoveal regions in the retina.

### Optical coherence tomography

Optical Coherence Tomography (OCT), a non-invasive imaging procedure, utilizes infrared light to create a cross-sectional image of the retina, and the accuracy of the retinal view generated by OCT is at least 10-15 microns (29). The images obtained by OCT can characterize the retina layers in the form of hypo- or hyper-reflective bands. OCT is a sensitive examining tool for GA evaluation as it provides a detailed characterization of retinal layers (3). This method can be used to visualize drusen deposits, choroidal neovascularization, detachment of RPE, and subretinal fluid (21, 30). The choroidal vascularity index, as determined by structural OCT analysis, can be used to predict GA progression (31). Reticular pseudo drusen (RPD) are located above the RPE and hence are distinguished from conventional drusen (32, 33). The presence of RPD is usually associated with the progression of atrophy to the foveal region. RPD cannot be assessed by clinical examination; however, it can be visualized by FAF and OCT (34). Due to the faster acquisition and the fine resolution of retinal images, OCT has emerged as a comfortable and preferred modality to assess GA lesions in patients (35). Technological advances in SD-OCT have improved the imaging performance and clinical usability of the technique, and improved visualization of photoreceptors, retinal pigment epithelium, and Bruch’s membrane may facilitate earlier detection and treatment of retinal diseases.

Based on the OCT findings and the anatomical layers affected, GA is classified into four categories: incomplete RPE and outer retinal atrophy (iRORA), complete RPE and outer retinal atrophy (cRORA), incomplete retinal atrophy, complete retinal atrophy (3, 36, 37). A region of choroidal signal hyper-transmission along with a corresponding zone of disruption or attenuation of the RPE layer and the presence of drusen and degenerated photoreceptors is defined as iRORA (38). The presence of these typical findings is a risk factor for the progression to cRORA, which is characterized by a region of hyper-transmission and the zone of disrupted or attenuated RPE of over 250 µm, with the presence of degenerated photoreceptors and an intact RPE (36–38).

## Molecular mechanisms underlying the pathophysiology of geographic atrophy

Several internal metabolic and oxidative stressors and external stressors such as advanced age and smoking are risk factors and trigger the pathogenesis of GA (11) (**Figure 1**). In early AMD, the macula has an abnormal RPE pigment distribution and drusen deposition in the space between the basal lamina of RPE and the inner collagenous layer of Bruch’s membrane (2). With age, these stressors induce damage to the RPE and cause the accumulation of extracellular deposits in the form of drusen (39, 40). Drusen particles are made up of lipids, proteins, cellular debris, β-amyloid deposits, apolipoproteins, iron, and zinc ions (41). Large or soft drusen with indistinct borders are a major risk factor for AMD progression. A2E was shown to increase the lysosomal pH of the RPE cells and thereby impair the ability of lysosomal degradation (42, 43). In addition, LF generates reactive oxygen species (ROS) upon photoinduction (44) that induces damage to the RPE cells (43). LF accumulation occurs over time resulting from the phagocytosis of membranous discs that are shed from retinal photoreceptors, and due to the inability of the RPE cells to degrade these granules. Excessive exposure to blue light was shown to damage RPE, while overexposure to white light induced photoreceptor cell damage. Activation of the inflammatory pathways in the presence of extrinsic and genetic risk factors contributes to GA pathogenesis.

**Figure 2:**
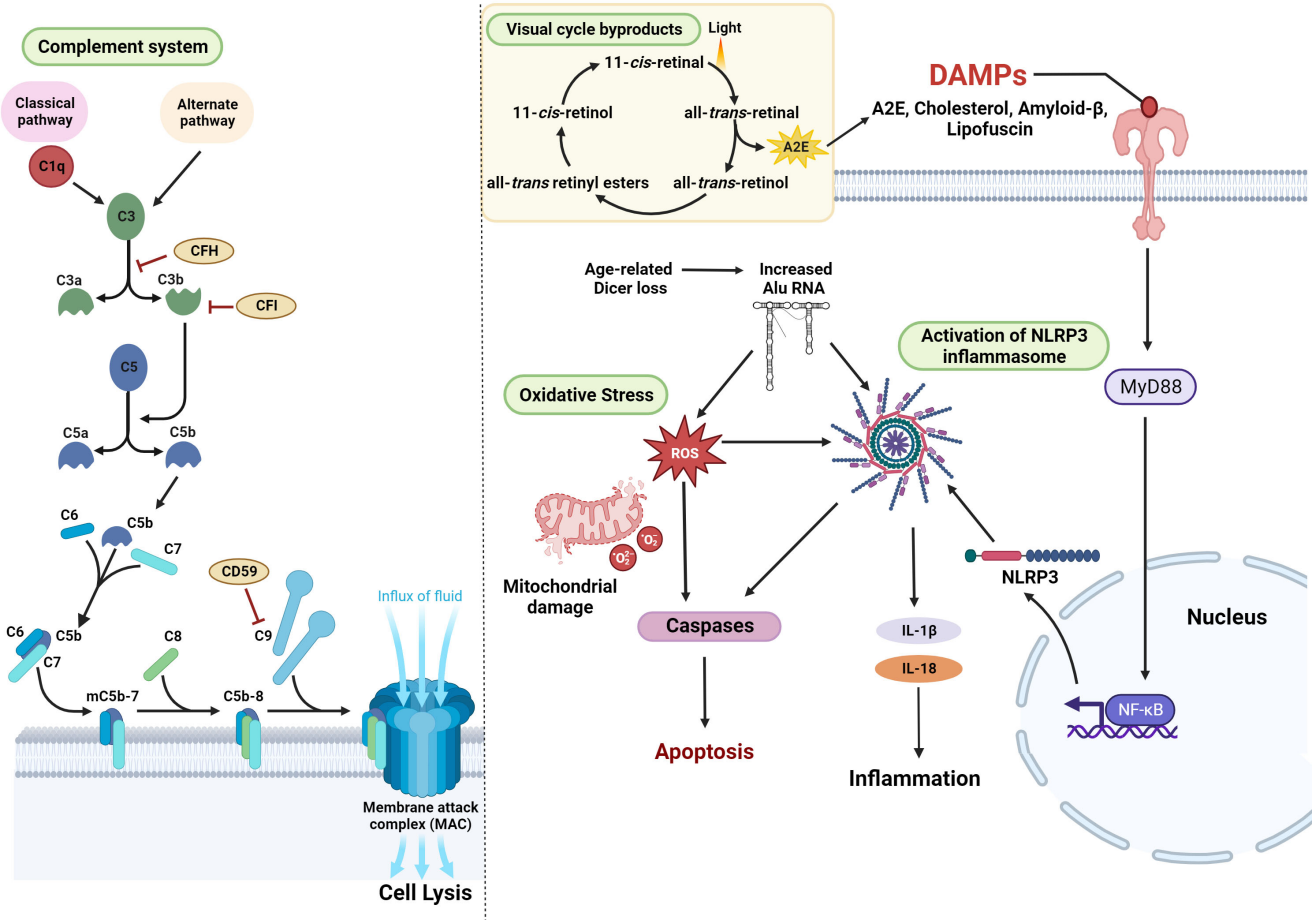
Cellular pathways associated with the development and progression of GA – Activation of the complement pathway, resulting in lytic cell death through MAC, is one of the major mechanisms associated with the pathology of AMD. Mitochondrial oxidative stress and the generation of ROS are also key contributing factors in the etiology of GA. In addition, damage-associated molecular patterns (DAMPs) such as cholesterol, amyloid-β Lipofuscin, or A2E (a byproduct of the visual cycle) activate Myd88 signaling and NFkB-induced transcription leading to the enhanced expression of NLRP3. Alternatively, age-related Dicer deficiency results in an abnormal increase in Alu RNA transcripts. Alu RNA-induced Myd88 signaling or the direct activation of NLRP3 results in inflammation, contributing to the disease pathogenesis (figure generated using bioRender.com).

Vitamin A (retinol), a cofactor in the phototransduction cycle, is a crucial component of the photopigment (11-*cis*-retinal). Absorption of a photon in the neural retinal outer segment releases retinaldehyde from the opsin protein. This results in the photoisomerization of 11-*cis*-retinal to all-*trans*-retinal, which subsequently results in the conversion of the visual pigment to the signaling form, meta-rhodopsin II, and transformation of light photons to electrical signals (45). Vitamin A is shuttled across the retina (between the photoreceptors and the RPE) as various isoforms: all-*trans*-retinol, all-*trans*-retinyl esters, all-*trans*-retinaldehyde, 11-*cis*-retinol, 11-*cis*-retinyl esters, 11-*cis*-retinaldehyde, and retinoic acid as a part of the visual cycle (46). Several biochemical pathways are involved in vitamin A transfer and storage in the RPE and transfer to the photoreceptors (47). During the visual cycle, a portion of this retinaldehyde condenses with phosphatidylethanolamine (PE) to form retinaldehyde-PE, leading to the formation of vitamin A dimer by-products, N-retinylidene-N-retinylethanolamine (A2E) (**Figure 2**), and all-*trans*-retinaldehyde dimer (ATR-dimer) (48). At the end of the visual cycle, specific enzymes carry out the regeneration of the active form of vitamin A (11-*cis*-retinal), the recycling of inactive forms, and the removal of toxic by-products (47). Accelerated dimerization of vitamin A is responsible for the formation of granular lipofuscin deposits in the eye, causing RPE atrophy (49). Abnormal accumulation of toxic dimers, such as A2E, causes protracted degeneration of the RPE and is associated with the etiology of AMD and Stargardt disease (49, 50).

### The complement pathway

Activation of complement cascade, a noncellular component of the innate immune system, is integral to the pathogenesis of GA (51). The complement system consists of over 30 circulating systemic proteins, which are associated with inflammation, opsonization, phagocytosis, and cell death and are responsible for the detection and clearance of pathogens (52). The activation of the complement system occurs via three pathways – the classical pathway, which is activated by antigen-antibody complexes, the lectin pathway, which is activated by the polysaccharides on microbes or the alternative pathway, which is activated by the cell surfaces of foreign pathogens (53). C1q is an initiating factor of the classical complement pathway, which is activated by components of drusen such as lysophospholipids (generated by lipase degradation of lipoproteins) and β-amyloid deposits (54, 55). Accumulation of C1q with age and inflammation induces retinal damage and is a key factor linked to the initiation and progression of GA (56). Initiation of the pathway ultimately results in the formation of multi-subunit serine protease complexes known as the C3 and C5 convertases, that act on the central complement proteins C3 and C5 (52). Cleavage of complement factor C3 into pro-inflammatory anaphylatoxin C3a and opsonin C3b induces inflammation and opsonizes the cells for phagocytosis. In AMD patients, components of the complement system C5, C3, and its fragments were detected in the subretinal space and the drusen particles (56, 57). C3 and its activation products were shown to induce multiple pathways, including inflammasome activation, recruitment of macrophages and microglia to the subretinal space, aberrant turnover of lysosomes and activation of intracellular C3 mechanisms within the RPE, such as the mTOR pathway (58–60). Complement factor B is a 93-kDa single-chain glycoprotein required for the activation of the alternative pathway. Association of factor B (FB) with protein C3b results in its cleavage by complement factor D to release an N-terminal FBa and the bound carboxyl-terminal serine proteinase FBb fragments to form the C3bBb C3 convertase (61). This convertase can cause further amplification of the complement pathway and the formation of MAC. Elevated plasma levels of activation fragments FBa and FBb, predominantly located in the vitreous, Bruch’s membrane, and choroidal vasculature, have been reported in patients with AMD (61, 62). The cleavage product C3b leads to the activation of C5 convertase, which acts on C5 to generate the pro-inflammatory anaphylatoxin C5a (which binds C5aR on immune cells) and the cleavage product C5b. Subsequently, C5b recruits C6, C7, and C8, and polymerization of 12-18 molecules of C9 forms C5b-9 to form a pore called the membrane attack complex (MAC). Accumulation of MAC beyond a certain threshold leads to disruption of the membrane and results in lytic cell death. CD59 is a glycoprotein that inhibits the polymerization of C9, preventing the formation of C5b-9 and MAC (63). The complement cascade is inactivated by regulatory factors like complement factors H and I (CFH and CFI). Negative regulation of the pathway by CFH results in the inactivation of C3b (iC3b), preventing the generation of the C5 convertase and thereby inhibiting the generation of MAC (**Figure 2**). RPE dysfunction results in a compromise in the blood-retinal barrier and allows leakage of serum proteins and components of the classical and alternative complement pathways into the retina from the underlying choriocapillaris, making it susceptible to the MAC (64). Studies in mice deficient in complement component receptors such as C3aR and C5aR demonstrated that these mice developed early and progressive retinal degeneration, suggesting the importance of this pathway in preserving retinal structure and function (65). In another independent study, C3 knockout mice exhibited severe photoreceptor loss, thickening of Bruch’s membrane, and high levels of retinal inflammation compared to control groups (66). In addition, reduced retinal function, thinning of retinal layers with age, and impaired signaling in the retinal INL and ONL were observed in complement knockout mice (67). These findings indicate that complement pathway components are critical for maintaining retinal homeostasis and integrity during the aging process. Therefore, it is imperative that the critical role of early complement factors in retinal biology should be carefully considered while developing therapies for AMD. Several GA-linked genetic risk factors pertaining to the complement cascade can cause dysregulation of this pathway, resulting in hyperactivation and inflammation, leading to retinal cell death, which is a characteristic feature of GA.

### Activation of the NLRP3 inflammasome

A multiprotein scaffold of the cellular stress sensor NLRP3 (nucleotide-binding domain, leucine-rich–containing family, pyrin domain–containing-3), adaptor ASC (apoptosis-associated speck-like protein), and the effector caspase 1 constitute the NLRP3 inflammasome (68). Activation of the inflammasome by damage-associated molecular patterns (DAMPs) like cholesterol crystals, lipofuscin, or the presence of drusen components such as Aβ-peptide 1–40 or C1q (68, 69) results in elevated interleukin (IL)-1b and IL-18 cytokine levels which mediate innate and adaptive immune pathways (68, 70–72).

Risk factors, such as aging, cause a buildup of cellular oxidative stress and lead to downregulation of the post-transcriptional expression of the miRNA processing enzyme, DICER1 (73). In GA, repetitive transposable elements of non-coding RNA, termed Alu RNA, were shown to accumulate in RPE due to a loss of DICER1 (74, 75). Alu RNA hinders the expression of ROS scavengers like superoxide dismutase 2 (SOD2) and endothelial nitric oxide synthase (eNOS) by directly intervening with their transcription and translation initiation (76). Alu RNA induces the priming of NLRP3 inflammasome via the pro-inflammatory transcription factor nuclear factor kappa B (NF-κB) pathway, promoting the transcription of inflammasome components. In GA, the RPE cell death is facilitated by apoptosis mediated by Caspase-3, which is triggered by MyD88 signaling via the IL-18 receptor (69, 74, 75) (**Figure 2**).

### Oxidative stress

Cigarette smoking was shown to be a major contributor leading to the development of AMD in at least 27% of the cases (77). Components in cigarette smoke such as hydroquinone (HQ) cause cellular oxidative damage, resulting in the generation of reactive oxygen species (ROS) (78). In addition, photoreceptor shedding, excessive light exposure, and aging also contribute to oxidative stress in the retina (79). Increased intracellular oxidative stress causes mitochondrial DNA (mtDNA) damage, oxidation of mitochondrial proteins and lipids, and structural damage to the mitochondria, subsequently resulting in mitochondrial dysfunction (**Figure 2**). In addition, mitochondrial ROS generation can also be mediated by Alu RNA via voltage-dependent anion channels (VDAC)-1 and -2, which are major channels at the outer mitochondrial membrane for the exchange of metabolites that can subsequently impair mitochondrial potential (80). Photoreceptors utilize glucose for glycolysis and produce large amounts of lactate, which is then transported to the RPE cells. The RPE cells subsequently use this lactate for energy production through oxidative phosphorylation by mitochondria (81). Mitochondrial oxidative stress was shown to increase the glycolytic metabolism in the RPE due to metabolic reprogramming, thereby leading to its dysfunction and ultimately resulting in the disruption of the photoreceptors.(82) The metabolic shift in RPE correlated with severe disruption of photoreceptor mitochondria involving downregulation of translocase of the outer mitochondrial membrane 20 (TOMM20) expression and reduced COXIII/β-actin levels, which are critical for maintaining mitochondrial morphology (82). Cytosolic mtDNA released due to mitochondrial damage causes activation of cyclic GMP-AMP synthase (cGAS)-driven type I interferon signaling (IFN), thereby resulting in NLRP3 inflammasome activation (83).

Retinoic acid receptor-related orphan receptor alpha (RORα) is a nuclear receptor that regulates inflammatory and cholesterol metabolism pathways (84). RORα also regulates lipid metabolism and lipoproteins, such as high-density lipoprotein, serum amyloid A, and apolipoprotein A1 (85). RORα-mediated transcriptional inhibition of the NFκB pathway inhibits TNF-α induced expression of inflammatory cytokines such as IL-6, IL-8, and COX-2 (86). Gene expression data and linkage analysis revealed RORα SNPs rs4335725 and rs12900948 are associated with AMD (87). In addition, RORα was shown to negatively regulate pathological neovascularization by modulating inflammatory response in the choroid/RPE complex (88, 89).

### Role of microglia and monocytes in AMD pathobiology

Retinal microglia maintain homeostasis by eliminating toxic waste, surveying the environment for damage, and mediating innate immune response (90). When exposed to genetic and environmental risk factors, microglia express pathogenic cytokines, recruit monocytes to sub-retinal space, and affect photoreceptor and RPE integrity (91). Under stress conditions, the hyperactive immune response triggered by infiltrated mononuclear phagocytes, such as microglia, macrophages, and monocytes, activate NF-κB signaling and result in the secretion of inflammatory cytokines, and contribute to retinal degeneration (92). Deficiency in microglial checkpoint genes such as *CX3CR1* (Chemokine Receptor-1) was implicated in lowering the threshold of microglial activation and inducing age-related infiltration of mononuclear phagocytes (92). *CX3CR1* SNPs were found to be associated with an increased incidence of AMD (93).

## Genetic factors associated with GA

**Figure 3:**
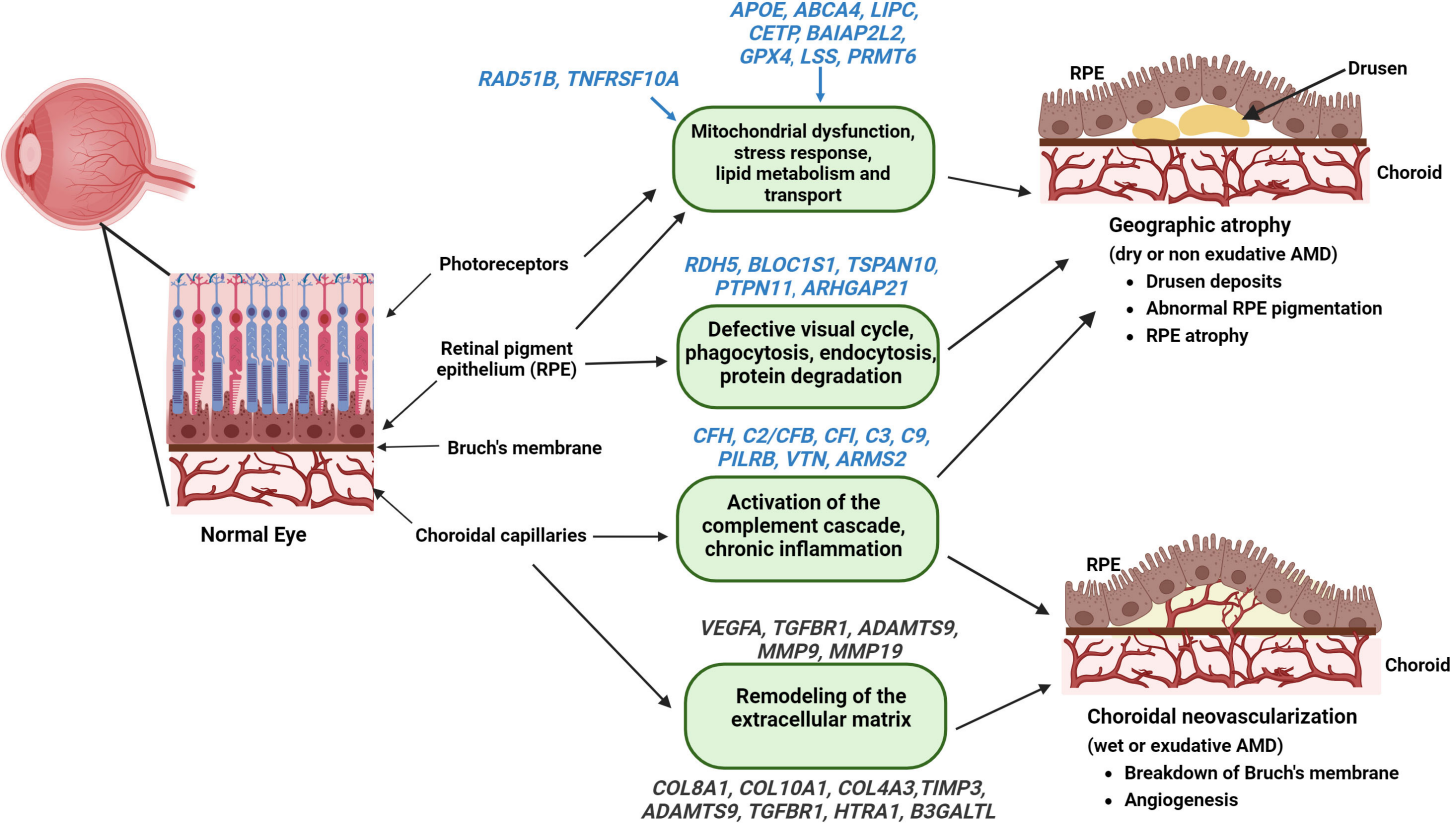
Genes associated with the pathophysiology of AMD – Identified genes (with established and putative roles) correlated with their functions that contribute to the pathogenesis of GA (highlighted in blue) and wet AMD (highlighted in Grey) are illustrated (figure generated using bioRender.com).

Genome-wide association studies indicate different genes associated with the prevalence of late AMD and progression of GA (60, 94, 95). The risk of AMD is amplified in the presence of genetic variants that regulate various facets of the complement system, including factors that negatively regulate complement activation, such as CFH and CFI. These factors are involved in convertase formation like complement factor B (CFB), C2 and C3, and C5b-9, which is a part of the MAC (96–99). A study comparing 3235 cases of GA and 10,749 cases with choroidal neovascularization identified 52 independently associated single nucleotide polymorphisms (SNPs) across 34 genetic loci (94). These SNPs were found in *CFH, C2/CFB, CFI, C3*, and *C9* genes involved in the complement pathway, *APOE, LIPC, CETP*, and *BAIAP2L2* genes involved in the lipid metabolism and transport pathways, *COL8A1, COL10A1, TIMP3, ADAMTS9, TGFBR1, HTRA1*, and *B3GALTL* genes involved in the remodeling and maintenance of extracellular matrix, *RAD51B* and *TNFRSF10A* genes involved DNA repair and cell survival, and *VEGFA, TGFBR1*, and *ADAMTS9* genes involved in angiogenesis (2) (**Figure 3**).

A common variant in the complement cascade gene, *CFH* (complement factor H), on chromosome 1 at the 1q31.3 locus, variant rs1061170 (p.Tyr402His), was strongly associated with an increased risk for developing AMD (96, 100–102). One *CFH* missense variant, rs121913059 (p.Arg1210Cys), was also linked to AMD (94). The p.Arg1210Cys and p.Tyr402His variants reduce the binding of *CFH*, resulting in reduced function, thereby preventing complement system inhibition in local chronic inflammation (103).

Among the identified lead variants associated with GA, the high-temperature requirement factor A1 (*HTRA*1) and the age-related maculopathy susceptibility 2 (*ARMS2*) are two tightly linked genes located on chromosome 10q26 (Chr10 locus). ARMS2 protein was shown to regulate complement activation by recruiting complement activator properdin, increasing C3b surface opsonization and phagocytosis. An SNP rs11200638 in the 10q26 genetic locus located at the promoter region of the *HTRA1* gene increased the expression of *HTRA1* in the RPE (104, 105). Two other polymorphisms in the 10q26 genetic locus, an *ARMS2* SNP variant Ala69Ser, (rs10490924), and an insertion/deletion polymorphism (del443ins54) in 3′UTR of *ARMS2* were identified to associate with AMD (94, 106). HTRAs are a conserved group of serine proteases containing a protease and a PDZ domain in the C-terminal region that function in substrate binding, oligomerization, and protein translocation (107). Substrates of HTRA1 include fibronectin, transforming growth factor Beta (TGF-β), clusterin, an inhibitor of angiogenesis thrombospondin (TSP1), and an extracellular matrix protein EFEMP1 (108) HTRA1 inhibits the canonical Wnt signaling pathway and modulates the TGF-β pathway. HTRA1 regulates angiogenesis by interacting with various members of the TGF-β family, such as TGF β1, TGF β2, activin, BMP4, and growth differentiation factor 5 (GDF5) and is associated with neovascular AMD (109). Aberrant accumulation of HTRA1 and its substrates, such as EFEMP1, form drusen-like deposits and activate the complement pathway (110). Overexpression of HTRA1 results in polypoidal lesions, the formation of subretinal deposits, and vascular abnormalities that are associated with AMD (111, 112). Oxidative stress, one of the major causative factors for AMD, induces HTRA1 expression in the RPE cells and increases cell senescence via the p38/MAPK pathway (113).

Other identified lead variants are *CETP, MMP9*, and *SYN3-TIMP3* loci, which demonstrated a significant difference between the AMD disease subtypes (94). In addition, the protein arginine methyltransferase 6 gene (*PRMT6*; chromosome 1p13.3) and the lanosterol synthase gene (*LSS*; chromosome 21q22.3) were proposed as the functionally relevant genes associated with the progression of GA (95). The variant rs42450006 upstream of *MMP9* was shown to be specifically associated with choroidal neovascularization but not with GA (94). Retinal transcriptome and expression quantitative trait loci (eQTL) analysis identified *PILRB/PILRA* (paired immunoglobin like type 2 receptor beta/alpha), B3GLCT (beta 3-glucosyltransferase), *BLOC1S1* (biogenesis of lysosome related organelles complex 1 subunit 1), *TMEM199* (transmembrane protein 199), and *TSPAN10* (tetraspanin 10) as additional putative causal genes for AMD (114, 115).

## Potential biomarkers for the detection of AMD

Retinal biomarkers can immensely contribute to the early detection of AMD and are evolving research areas research. Imaging biomarkers such as drusen characteristics, size, and area of drusen and pigmentary changes identified by color fundus photography can be used to classify stages of AMD (116). The most widely used imaging technique is OCT, which can be used to detect biomarkers such as drusen volume, hyper-reflective foci, hyper-transmission defects, reticular pseudo drusen, and iRORA (116). In the context of GA, FAF patterns are extremely helpful markers for the evaluation of atrophic areas and disease progression (117). Multifocal electroretinography (mfERG) measures photoreceptor signaling in response to a light stimulus using contact electrodes in the cornea (118). The functional areas of the retina and their sensitivity can be mapped by this technique by varying the intensity of the light stimulus and thereby can be used for early detection of AMD (119). Assessment of visual function by testing for visual acuity, dark adaptation, contrast sensitivity, and evaluation of retinal sensitivity by microperimetry can be specific and significant markers that indicate different stages of disease progression (19, 120).

Studies indicate a strong association between the family history of AMD and the incidence of the disease. The risk of AMD increases 4-fold for those with a family history and nearly 27 times for those with an affected parent (121). Therefore, the incorporation of comprehensive natural history studies and identification of associated genetic biomarkers will aid in early detection. SNPs in genes with specific functions in the retina, such as *ABCA4* and *TIMP-3*, genes pertaining to complement pathway, such as *CFI*, *C3*, and *CFH*, genes related to lipid metabolism, such as *ApoE*, and immune function related genes, such as *ARMS2/HTRA1* can be potential genetic biomarkers (122).

**Figure 4:**
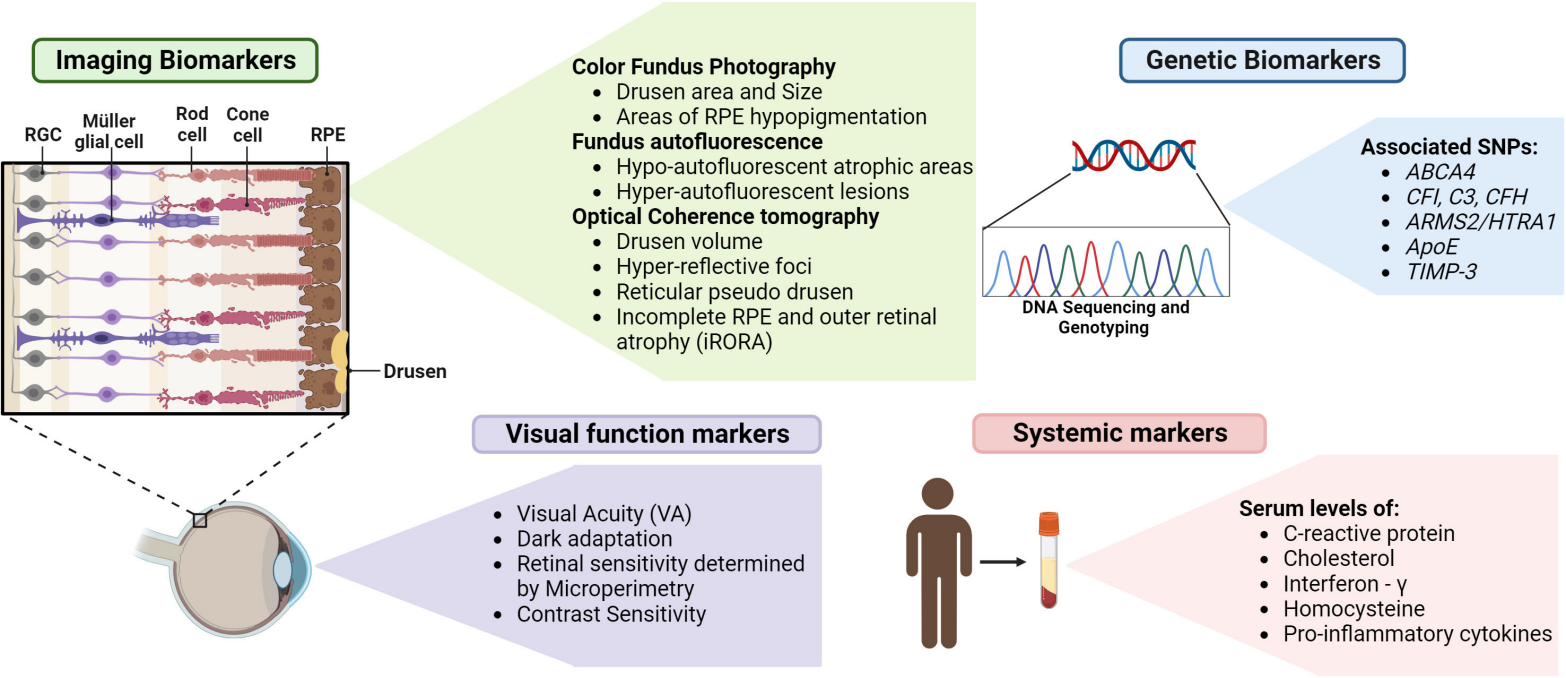
Biomarkers for detection of GA – Various biomarkers indicated above can assist in the early detection, evaluation of GA progression and treatment outcomes (figure generated using bioRender.com).

Identifying serum biomarkers for AMD can aid in monitoring disease progression and treatment response. Evaluation of levels of C-reactive protein (CRP), cholesterol, Interferon-γ, Homocysteine, and proinflammatory cytokines such as IL-8 can be indicative of AMD risk and can be developed as screening tools (122). The various biomarkers that can be used for early diagnosis are summarized in **Figure 4**.

## Treatment of GA and ongoing clinical trials

**Figure 5:**
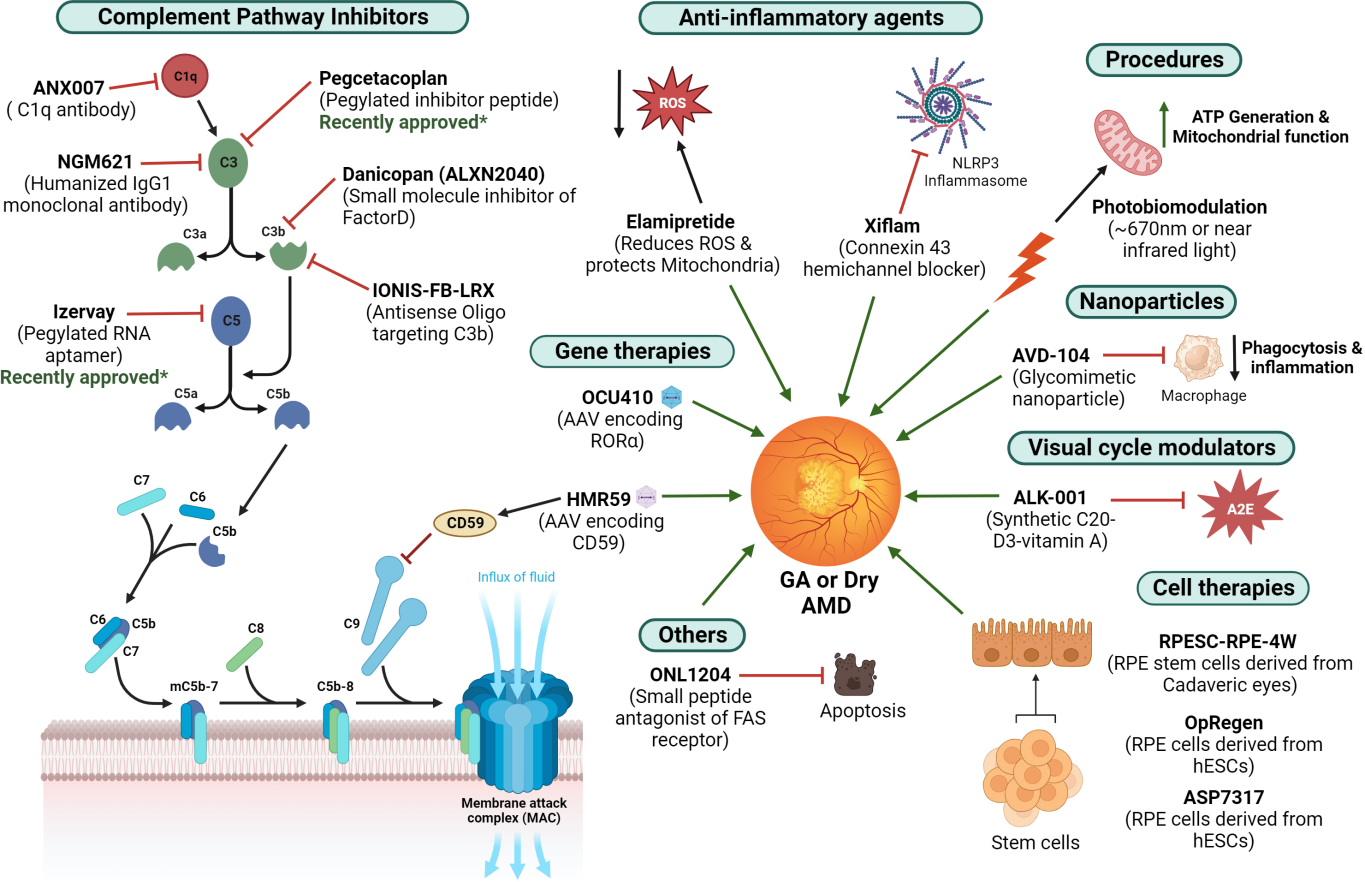
Current treatment strategies for GA – Different therapeutic agents under ongoing clinical trials that are being investigated for the treatment of GA are illustrated (figure generated using bioRender.com). The therapies approved recently are also indicated.

Despite an increased understanding of the underlying risk factors and the inflammatory pathways contributing to the pathophysiology of GA, limited treatment options are available to combat the disease. Several therapeutic strategies that target the complement pathway and reduce inflammation that are under clinical trials for the treatment of GA are listed in **Figure 5**.

### Inhibition of the complement pathway

#### C3 inhibition

Pegcetacoplan is a synthetic pegylated complement C3 inhibitor peptide that was developed by Apellis Pharmaceuticals. The phase III OAKS and DERBY studies (NCT03525600, NCT03525613) in which the patients with GA were given both monthly and bimonthly doses of pegcetacoplan demonstrated a clinically significant reduction in GA lesion growth. Recently, intravitreal pegcetacoplan (Syfovre; Apellis Pharmaceuticals, Inc.) was approved by the FDA for patients with GA. However, in clinical trials, by month 24, administration of Syfovre was associated with a higher rate of progression to neovascular AMD or choroidal neovascularization (12% when administered monthly, 7% when administered every other month).

NGM621 is a humanized immunoglobulin G1 (IgG1) monoclonal antibody that aims to inhibit C3 activity that is developed by NGM Biopharmaceuticals. CATALINA Phase II trial (NTC04465955) evaluated NGM621 for treatment of GA and failed to meet its primary endpoint - the change in GA lesion area as measured by FAF over the 52 weeks of treatment. Further update on the trial results is awaited as the evaluation of specified secondary endpoints and *post-hoc* analyses are underway.

#### C5 inhibition

Another component of the complement system being targeted is C5. An Avacincaptad Pegol (ACP, a pegylated RNA aptamer developed by Iveric Bio, Inc.), which binds and inhibits C5, was under clinical trials. (123) Phase II/III GATHER1 (n=286, NCT02686658), which included 286 patients, and Phase III GATHER2, including a larger cohort of 448 patients (NCT04435366), which administered 2 mg of the avacincaptad pegol or sham monthly were also conducted. Data from GATHER2 showed that treated patients had a 14.3% reduction in the average rate of GA area growth over 12 months. FDA approved this treatment recently for patients with GA and is now marketed as Izervay. However, by month 12, the use of Izervay was linked to increased rates of wet AMD or choroidal neovascularization (7% when administered monthly and 4% in the sham group) in clinical trials.

#### C1q inhibition

Inhibiting C1q activation prevents the classical complement activation cascade (which includes C4, C3, and C5), implicated in retinal tissue damage. (124) Pharmacological inhibition of C1q was shown to preserve the function of photoreceptor cells and prevent their progressive loss, even if the photooxidative damage was induced before the administration of the inhibitor. (125) A high-affinity antibody Fab fragment binding to C1q, ANX007, was tested by intravitreal (IVT) administration in cynomolgus monkeys in a study, which was reported to be well tolerated and resulted in near complete C1q inhibition (124). A Phase 2 clinical trial of ANX007, ARCHER study (NCT04656561), comparing the safety and efficacy of ANX007 administered either monthly or every other month, was conducted in 270 patients with GA. Results from the 12-month treatment period demonstrated a statistically significant, dose-dependent preservation of visual function in the patients treated monthly. However, the rate of lesion growth was not reduced by this treatment (Annexonbio.com). Based on the results of this trial, ANX007 is still being developed as a potential therapy for the preservation of visual acuity in patients with GA.

#### Factor D inhibition

Factor D is an essential enzyme that cleaves factor B bound to C3b and mediates the activation of the alternate complement pathway. Danicopan (ALXN2040) is a small molecule inhibitor that has high affinity binding to factor D and can thereby inhibit alternate pathway activation, ultimately blocking the inflammatory response and cell lysis (126). Alexion Pharmaceuticals is conducting a dose-finding phase II clinical trial for oral Danicopan for GA patients without foveal involvement (NCT05019521).

#### Gene therapy to inhibit the complement pathway

CD59 is a glycosylphosphatidylinositol (GPI)-anchored membrane protein that prevents the incorporation of C9 into the C5b-8 complex, thereby inhibiting the action of the MAC on cell membranes (127). Hemera Biosciences developed HMR59 (AAVCAGsCD59), which is an intravitreally administered adeno-associated virus 2 (AAV2) vector that delivers the soluble form of CD59 (sCD59). HMR-1001 (NCT03144999), a phase I safety and efficacy study, reported that patients with GA showed a 23% reduction in GA progression when administered HMR59. However, 27% of the patients given HMR59 reported mild vitritis and uveitis, requiring 6–8 weeks of anti-inflammatory therapy (128).

GT005, developed by Gyroscope Therapeutics, is a recombinant non-replicating AAV vector encoding human CFI, an inhibitor of the complement system. Preliminary results of phase I/II clinical trial, FOCUS (NCT03846193) delivered GT005 via a subretinal injection in 28 patients with GA reported a significant (122%) increase in CFI levels and up to a 46% reduction in the levels of key C3 breakdown proteins associated with complement activation compared to baseline levels (Gyroscopetx.com) (128). Other ongoing phase II clinical trials for GT005 include EXPLORE (NCT04437368), to evaluate patients with GA who have low levels or rare variants of CFI, and HORIZON (NCT04566445), which evaluated a broader group of patients with GA secondary to AMD. The Explore and Horizon trials were terminated recently as they did not meet the efficacy outcome.

#### Antisense oligonucleotides to inhibit the alternate complement pathway

Complement factor B is required for the activation of the alternative complement pathway. In a study, second-generation ASOs targeting factor B were administered subcutaneously in healthy mice or monkeys, resulting in a significant decrease in both ocular and plasma factor B protein levels (129). IONIS-FB-LRX, developed by Ionis Pharmaceuticals and Roche, is a 2’-O-methoxyethyl (2’MOE) second-generation ASO (20 bp) conjugated to an N- acetyl galactosamine (GalNAc) ligand (for efficient liver targeting), is an antisense inhibitor of complement factor B (128). The GOLDEN STUDY is a phase 2 clinical trial assessing the safety and efficacy of IONIS-FB-LRX for patients with GA (NCT03815825).

### Anti- oxidative agents

Elamipretide, developed by Stealth Biotherapeutics, is a small tetrapeptide (D-Arg-dimethylTyr-Lys-Phe-NH2) that reduces the production of ROS and protects mitochondrial function. The phase II clinical trial, ReCLAIM-2 study (NCT03891875) in 180 patients who received a daily 40mg subcutaneous injection of Elamipretide for 48 weeks failed to meet its primary endpoints—change in low-luminance visual acuity (LLVA) and GA lesion size. However, the latest data from study participants was promising and demonstrated >2-line improvement in LLVA, so the program is being continued by Stealth Biotherapeutics.

### Anti- inflammatory agents

NLRP3 inflammasome complex is activated and assembled by Connexin43 hemichannel-mediated ATP release, and inhibition of connexin 43 hemichannels reduces inflammation in cells (130). Oral connexin 43 hemichannel blocker, tonabersat (Xiflam) by InflammX, is under Phase IIb clinical trials for treating intermediate dry AMD and GA; however, no trials are registered at ClinicalTrials.gov.

Retinoic acid-related orphan receptor α (RORα) is a nuclear hormone receptor involved in the suppression of inflammatory cytokine expression and inhibition of complement factor activation (86, 88, 131). OCU410, which delivers RORα, is being developed as a modifier gene therapy for GA by Ocugen. A recent study demonstrated that OCU410 treatment decreased drusen-like deposition in Abca4-/- mouse retinas and improved retinal function (131).

### Cell therapy for GA

Luxa biotechnology is investigating the efficacy of transplanting allogeneic retinal pigment epithelium stem cell (RPESC)-derived RPE cells isolated from the RPE layer of human cadaveric eyes (RPESC-RPE-4W) in the macular region of the eye. Phase I/II clinical trial evaluating the safety and tolerability of RPESC-RPE-4W as therapy for dry AMD or GA is ongoing (NCT04627428).

OpRegen, developed by Lineage Cell Therapeutics in partnership with Genentech/Roche, uses human embryonic stem cell (hESC) derived RPE cells. The Phase I/II trial (NCT02286089) administered RPE cells sub-retinally as a cell suspension in 24 patients with dry AMD. The trial reported no cases of rejection, acute or delayed intraocular inflammation, or sustained increases in intraocular pressure after OpRegen transplantation. Currently, a Phase IIa clinical trial (NCT05626114) to further assess the safety of subretinal surgical delivery and to test the preliminary activity of OpRegen in participants with GA is underway.

ASP7317 (RPE cell program, MA09-hRPE) is a cell therapy developed by Astellas Institute for Regenerative Medicine. A phase Ib clinical trial for subretinal administration of RPE cells derived from hESCs to evaluate their efficacy in slowing or reversing the atrophic lesion growth in dry AMD is under progress (NCT03178149).

### Modulators of the visual cycle

Fenretinide or 4-HPR (N-(4-hydroxyphenyl) retinamide) is a synthetic derivative of vitamin A which competes with retinol and binds to retinol-binding protein (RBP) (132). The formation of the RBP-Fenretinide complex results in the reduction in retinol delivery and decreased accumulation of toxic visual cycle byproducts. A phase II study which administered different doses of oral Fenretinide in GA patients established that there was a reduction in GA lesion growth rate and neovascularization in treated subjects (133).

A synthetic vitamin A with deuterium at the C20 position (C20-D3-vitamin A or ALK-001) was shown to slow down the formation of toxic vitamin A dimers by 4-5 fold (46, 48). The oral formulation of ALK-001 is currently under phase 3 clinical trial (NCT03845582) for the treatment of GA (134).

### Other treatments

Belite Bio developed Tinlarebant, an oral, small-molecule antagonist of retinol-binding protein 4 (RBP4). A phase I trial (NCT03735810) in 71 patients confirmed the safety, tolerability, and achievement of a potential therapeutic level of the drug. A phase III trial is being planned for this drug for patients with GA.

ONL1204, a small peptide antagonist of fragment apoptosis stimulator (FAS) receptor, which can inhibit Fas receptor-mediated apoptosis, is under development by ONL therapeutics. In preclinical AMD models, ONL1204 was shown to protect the RPE cells (Cera.org.au). Intravitreal administration of ONL1204 is under phase I clinical trial for patients with GA (NCT04744662).

A glycomimetic sialic-acid coated nanoparticle, AVD-104, can specifically bind to receptors on activated macrophages and repolarize them to a resting state, thereby hindering their phagocytic function (avicedarx.com). AVD-104, which was also shown to inhibit the complement cascade and reduce the inflammatory damage in preclinical retinal models, is being investigated by Aviceda therapeutics for the treatment of GA. A phase II clinical trial was recently initiated to assess the efficacy and pharmacokinetics of AVD-104 in GA patients via intravitreal administration (NCT05839041).

Cholesterol-lowering statins can be efficacious for clearing up lipid debris in patients with AMD (135). In a study with high-risk AMD patients, high-dose atorvastatin (80mg) was shown to reduce the accumulation of lipid deposits, improve visual acuity, and slow the progression to advanced disease (136). Druslov Therapeutics is planning a Phase II clinical trial for Ocustatin.

Toxic oxidation of polyunsaturated fatty acids in photoreceptor membranes, such as docosahexaenoic acid (DHA), is one of the contributing factors for retinal degeneration. Deuterated DHA is resistant to lipid peroxidation and can have protective effects against retinal degeneration (137). Biojiva is exploring this as a possible therapy for dry AMD.

During aging, reduced expression of the *ELOVL2* (Elongation of very long chain fatty acids-Like 2) gene results in a decline in long and very long-chain polyunsaturated fatty acids (PUFA’s), which is associated with AMD pathogenesis (138). Visgenx Inc. is planning subretinal delivery of VGX-0111 (carrying an *ELOVL2* transgene) as a treatment for dry AMD.

A large Age-Related Eye Disease Study (AREDS) reported that daily intake of certain nutritional vitamins and minerals lowered the rate of drusen formation and decreased the risk of wet AMD and vision loss. These are marketed as AREDS 2 supplements and are available for use in patients with a high risk of developing AMD.

### Procedures

Exposing the retina to low-intensity light (~670 nm or near-infrared light) was shown to improve retinal mitochondrial function due to the absorption of light by cytochrome C oxidase, which results in its increased expression and enhances cellular respiration and ATP generation (139, 140). This process, also known as photobiomodulation, is being investigated as a potential therapy for GA. The Lightsite I clinical trial utilizing the LumiThera LT 300 Light Delivery System, which exposes patients with dry AMD to wavelengths of light ranging from 500-1000 nm, was shown to significantly reduce drusen thickness and volume and improve clinical outcomes (141) (NCT02725762). The Lightsite III clinical trial (NCT04065490) utilizing the Valeda Light Delivery System, which delivers 590, 660, and 850 nm of light to the study eye of patients with dry AMD, met its primary efficacy endpoint and demonstrated improved anatomical and clinical outcomes (142).

## Future directions

Currently, there are two FDA-approved treatments for GA that target the complement pathway: Pegcetacoplan and Izervay. While these complement inhibitors have been approved and show promise to slow down lesion growth, their efficacy is limited, and they do not work for all patients. Also, these treatments are not the cure and only slow the progression of the disease. These treatments require frequent visits to the clinic and can be uncomfortable for patients because of multiple frequent administration during treatments. No therapy is currently able to repair the impaired retinal pigment epithelium (RPE) and outer retinal layers that are affected by GA.

In summary, the current treatment options for GA have limitations, including the lack of a cure, limited efficacy, the requirement for frequent administration, unknown factors underlying the disease, and the inability to repair the damage caused by GA. Extensive research has contributed to new GA classification based on advanced imaging techniques and phenotypic characteristics. Understanding and identifying genetic predispositions for diagnosis and designing specific treatment approaches is imperative. Promising therapies targeting multiple signaling pathways are underway to establish different strategies to treat the disease through gene therapy, stem cell therapy, and neuroprotective agents.

## Author contributions

KR: Writing – original draft, Writing – review & editing. FD: Writing – review & editing. AU: Writing – review & editing.
